# Radiation dose ≥54 Gy and CA 19–9 response are associated with improved survival for unresectable, non-metastatic pancreatic cancer treated with chemoradiation

**DOI:** 10.1186/1748-717X-7-156

**Published:** 2012-09-13

**Authors:** Daniel W Golden, Caroline J Novak, Bruce D Minsky, Stanley L Liauw

**Affiliations:** 1 Department of Radiation and Cellular Oncology, University of Chicago, 5758 South Maryland Avenue Mail Code 9006, Chicago, IL 60637, USA

**Keywords:** Pancreas cancer, Radiotherapy dosage, Chemotherapy, CA-19-9 antigen

## Abstract

**Background:**

Unresectable pancreatic cancer (UPC) has low survival. With improving staging techniques and systemic therapy, local control in patients without metastatic disease may have increasing importance. We investigated whether the radiation dose used in chemoradiation (CRT) as definitive treatment for UPC and the CA 19–9 response to therapy have an impact on overall survival (OS).

**Methods:**

From 1997–2009 46 patients were treated with CRT for non-metastatic UPC. Median prescribed RT dose was 54 Gy (range 50.4-59.4 Gy). All patients received concurrent chemotherapy (41: 5-fluorouracil, 5: other) and 24 received adjuvant chemotherapy.

**Results:**

41 patients were inoperable due to T4 disease and 5 patients with T3 disease were medically inoperable. Five patients did not complete CRT due to progressive disease or treatment-related toxicity (median RT dose 43.2 Gy). Overall, 42 patients were dead of disease at the time of last follow-up. The median and 12 month OS were 8.8 months and 35%, respectively. By univariate analysis, minimum CA 19–9 post-CRT <90 U/mL was favorably associated with OS (12.3 versus 8.8 months, p = 0.012). Radiotherapy dose ≥54 Gy trended towards improved OS (11.3 versus 6.8 months, p = 0.089). By multivariable analysis, a delivered RT dose of ≥54 Gy (HR 0.47, p = 0.028) and minimum CA 19–9 post-CRT of <90 U/mL (HR 0.35, p = 0.008) were associated with OS.

**Conclusions:**

CRT as definitive treatment for UPC had low survival. However, our retrospective data suggest that patients treated to ≥54 Gy or observed to have a minimum post-CRT CA 19–9 <90 U/mL had improved likelihood of long-term survival.

## Background

Pancreatic cancer is the fourth leading cause of cancer-related mortality in the United States with 43,140 new cases and 36,800 deaths estimated in 2010 [1]. Approximately 50% of patients diagnosed with pancreatic cancer have distant metastases at the time of diagnosis. Of the remaining patients with local-regional disease, approximately 70% have tumors that are unresectable [2]. Historically, the prognosis for patients with unresectable pancreatic cancer is low and the median survival with chemotherapy or chemoradiation (CRT) is between 8 to 12 months. Currently, treatment options for unresectable pancreatic cancer include CRT, chemotherapy alone, and chemotherapy followed by CRT [3].

Chemotherapy alone or with concurrent radiotherapy is a commonly accepted standard of care for unresectable pancreatic cancer because of the high risk of occult distant metastases. However, the role of radiotherapy in the treatment of unresectable pancreatic cancer is less clear [4]. Radiotherapy as a component of treatment for patients with unresectable pancreatic cancer is disputed on the assumption that patients with pancreatic cancer die of distant metastases. However, two autopsy studies have shown that approximately 30% of deaths in patients with pancreatic cancer are due to locally progressive disease alone [5,6]. Retrospective [7] and prospective studies [8,9] support the hypothesis that CRT can improve survival by decreasing local failure, although other studies are not confirmatory [4].

At the University of Chicago, patients with unresectable, non-metastatic pancreatic cancer are often treated with CRT. We report our institutional experience using CRT as definitive treatment for unresectable pancreatic cancer by analyzing patient and treatment related factors in relation to overall survival.

## Methods

Between January 1997 and January 2010, 46 consecutive patients were treated with definitive CRT for non-metastatic unresectable pancreatic cancer at the University of Chicago Medical Center. Patient characteristics are summarized in Table 1. The University of Chicago Institutional Review Board reviewed and approved this retrospective analysis. Patients were included if they were treated with radiotherapy for unresectable pancreatic cancer. Nine patients were borderline resectable on staging studies, but at the time of exploratory laparotomy were deemed unresectable. Patients were not excluded from the analysis if the treatment intent was curative but radiotherapy was stopped before reaching the prescribed dose either for toxicity or progression of disease.

**Table 1:** Patient and treatment characteristics stratified by radiotherapy dose < or ≥ 54 Gy

		**All**	**<54 Gy**	**≥54 Gy**	**p value**
Number of patients		46	21	25	
Age at start of CRT, years (Median)		61	63	59	0.089
BMI kg/m2 (Median)		26.5	26.8	25.9	0.757
Exploratory laparotomy	No	37	16	21	
	Yes	9	5	4	0.506
T classification*	T3	5	2	3	
	T4	41	19	22	0.788
N classification	N0	24	12	12	
	N1	22	9	13	0.536
Histology	Adenocarcinoma	43	21	22	
	Other	3	0	3	0.101
IMRT	No	16	10	6	
	Yes	30	11	19	0.094

*T4 classification includes patients unresectable at exploratory laparotomy.

(BMI = body mass index; CRT = chemoradiation; IMRT = intensity modulated radiotherapy).

### Pretreatment evaluation

Patients were initially evaluated by a medical, surgical, and radiation oncologist and their cases were discussed at multidisciplinary tumor board. Patients were defined as unresectable based on imaging including CT, MRI, and/or endoscopic ultrasound (n = 32) or intraoperative exploration (n = 9) using the National Comprehensive Cancer Network guidelines [10]. Five patients had T3 disease but were medically inoperable. One patient with unresectable disease who was included in this analysis was locally recurrent with unresectable disease 10 months after previous resection. A total of 40 patients had a pre-CRT CA 19–9 level. The median time from tissue diagnosis to the start of radiation therapy was 38 days. At least one post-treatment CA 19–9 level was available in 39 patients.

### Treatment

Table 1 summarizes the patient and treatment characteristics for the groups treated with < or ≥54 Gy. None of the patient characteristic or treatment parameters were significantly different (p<0.05) between the low and high dose groups and all subsequent analyses were done on the entire cohort. Treatment characteristics are summarized in Table 2. Overall, 46 patients were treated with CRT for non-metastatic unresectable pancreatic adenocarcinoma. For simulation and treatment, patients were immobilized with upper and lower cradles and a CT simulation scan was obtained with 3 mm cuts. The median prescribed external beam RT dose was 54 Gy (range 50.4-59.4). Five patients did not complete CRT at a median RT dose of 43.2 Gy due to progressive disease (n = 2) or treatment-related toxicity (n = 3).

**Table 2:** Treatment characteristics

Prescribed RT dose, Gy (median; range)		54; 50.4 – 59.4
Delivered RT dose, Gy (median; range)		54; 18 – 59.4
Technique	3D-CRT	16
	IMRT	30
Initial RT dose, Gy (median; range)		45; 45 – 50.4
Boost RT dose, Gy (median; range)		9; 5.4 – 14.4
Respiratory Gating	Yes	6
	No	40
Concurrent chemotherapy	5-fluorouracil	41
	Capecitabine	2
	Motexafin gadolinium	2
	Gemcitabine	1

Treatment techniques and approaches were based on physician preference. Of the 46 patients, 30 (65%) patients were treated with step-and-shoot IMRT. The majority of patients treated with IMRT received sequential treatment of 45 Gy to the elective nodal region and primary tumor, followed by a boost to the primary tumor and involved nodes only. Three patients were treated with a simultaneous integrated boost of 55 Gy (2.2 Gy/fraction) to the primary mass, and 45 Gy (1.8 Gy/fraction) to the elective nodal region in 25 fractions. Dose constraints for the whole liver (V30 <65%), both kidneys (V20 <50%), and spinal cord (maximum dose <45 Gy) were used for treatment planning. Patients treated with 3D-conformal RT were generally treated with a four-field technique with AP, PA, and opposed lateral beams using a combination of 6 MV and 18 MV photon beams with custom blocks and wedges as necessary to increase dose homogeneity and spare normal tissues and organs at risk. The initial treatment volume included the gross tumor volume (GTV) and peripheral draining lymphatics. Boost volumes included the pancreatic tumor and grossly involved lymph nodes. The GTV to clinical target volume expansion was usually 1 cm except into bone or muscle and an additional 1 cm was added for the planning target volume expansion. 6 MV photon beams were used for all IMRT treatments. Two patients received neoadjuvant chemotherapy. All patients received concurrent chemotherapy (5-fluorouracil continuous infusion n = 38, 5-fluorouracil bolus n = 3, capecitabine n = 2, gemcitabine n = 1, motexafin gadolinium n = 2). Typical chemotherapy doses were either continuous venous infusion 5-fluorouracil 150–200 mg/m^2^/day for 96 hours every week, bolus 5-fluorouracil 500 mg/m2/day for 3 days during the first and last week of radiation, capecitabine 1500 mg/m^2^ by mouth twice daily throughout radiation therapy, gemcitabine twice weekly at 40 mg/m^2^/day throughout radiation therapy, and motexafin/gadolinium 2.9 mg/kg every Monday, Wednesday, and Friday throughout radiation therapy. 13 of the patients reported were enrolled on a randomized, multi-institutional trial investigating TNFerade. The radiotherapy dose for this trial was 50.4 Gy. In addition to infusional 5-fluorouracil, 9 of the patients received weekly intratumoral injections of TNFerade, a replication deficient adenoviral vector used to deliver tumor necrosis factor-α to the tumor cells. With the exception of the 13 patients enrolled on the TNFerade trial the selection of radiation dose was dependent on physician preference.

Adjuvant chemotherapy was given at the discretion of the treating medical oncologist. 24 patients received adjuvant chemotherapy (gemcitabine n = 20, 5-fluorouracil n = 2, irinotecan/taxotere n = 1, and immunotherapy n = 1). Nine patients did not receive adjuvant chemotherapy and adjuvant chemotherapy records were unavailable for 13 patients.

### Follow-up

Patients were scheduled for follow-up at least every 3 to 4 months for the first 2 years. Computed tomography scans of the chest, abdomen, and pelvis were obtained prior to these visits to follow disease status. A CA 19–9 measurement generally was drawn at each follow-up. The median time to the first CA 19–9 measurement following CRT was 34 days (range 3–347). Chemotherapy was given at the medical oncologist’s discretion. Therapy at the time of progression was per the treating medical oncologist.

### Endpoints

The primary endpoint of this study was overall survival. Overall survival was determined by reviewing the social security death index and hospital records. Secondary endpoints included local and distant failure. Local and distant failures were defined radiographically.

### Statistical analysis

Overall survival time was calculated from the first day of radiotherapy. Overall survival was censored at the date of last follow-up recorded (clinic visit, radiographic study, or laboratory value) if the patient remained alive. The two-sided Student’s *t*-test was used to compare the differences in means. Differences in proportions were calculated using the chi-square test. Survival probabilities were estimated using the Kaplan-Meier method. The log-rank test was used to analyze survival differences between groups. Only variables trending towards significance (p < 0.10) on univariate analysis were tested in multivariable analysis. Cox proportional-hazards modeling was used to examine the effect of radiotherapy dose, CA 19–9 parameters, and other parameters on overall survival. Stata v12.1 (StataCorp, College Station, Texas) was used for all statistical analysis.

## Results

At the time of analysis, 42 of 46 patients were dead of disease. The group included 19 men and 27 women. Tumors were located in the head (n = 31), body (n = 11), tail (n = 1), head/body (n = 1), and body/tail (n = 2). All but three patients had adenocarcinoma. The other histologies included acinar cell carcinoma, ductal carcinoma, and poorly differentiated carcinoma. The median follow-up was 8.7 months for all patients, and 33.0 months for surviving or censored patients. A median of 38 days elapsed between pathologic diagnosis and the start of RT. There was no correlation between time from biopsy to start of CRT and overall survival (p = 0.351). Median and 12 month overall survival from the start of CRT were 8.8 months and 35.0% (95% CI 21.4 – 48.9), respectively. 12 month freedom from local failure and distant failure were 52% and 30%, respectively.

Pre- and post-treatment CA 19–9 parameters were evaluated. The median pre-CRT CA 19–9 was 343 U/mL (range 0–18,955 U/mL). Because CA 19–9 levels can be artificially elevated in the setting of biliary obstruction, the total bilirubin level at the start of CRT was reviewed. Total bilirubin was normal (<1.2 mg/dL) for all but eight patients at the start of CRT. Only two patients had a total bilirubin >3 mg/dL the upper limit of normal. A total of 15 patients had a pre-CRT CA 19–9 ≤90 U/mL, and 24 had a pre-CRT CA 19–9 >90 U/mL. The median post-CRT CA 19–9 minimum was 89 U/mL (range 0–25,744 U/mL). In patients who had a post-treatment CA 19–9 value (n = 39), the median time to CA 19–9 minimum value was 66 days (range 3–1,313 days).

Patient characteristics and treatment parameters were analyzed in relation to overall survival with univariate analysis (Table 3). The only prognostic factor significantly associated with overall survival on univariate analysis was CA 19–9 post-CRT minimum <90 U/mL (≥90 U/mL 8.8 months versus <90 U/mL 12.3 months, p = 0.012). Delivered radiotherapy dose ≥54 Gy trended towards improved overall survival (≥54 Gy 11.3 months versus <54 Gy 6.8 months, p = 0.089). There was no difference in overall survival by tumor location, T classification, N classification, histology, pre-CRT CA19-9, treatment on protocol, or treatment with TNFerade.

**Table 3:** Univariate analysis of patient and treatment related factors in relation to overall survival

		**Median OS (months)**	**p value**
Age	<60	8.8	
	≥60	7.0	0.778
T classification	T3	4.9	
	T4	8.9	0.120
N classification	N0	8.8	
	N1	8.9	0.543
Exploratory laparotomy	No	8.9	
	Yes	8.5	0.550
Neoadjuvant chemotherapy	No	8.8	
	Yes	6.6	0.814
RT dose delivered	<54 Gy	6.8	
	≥54 Gy	11.3	0.089
RT modality	IMRT	8.8	
	3D	8.5	0.275
Protocol	On	8.5	
	Off	9.0	0.123
TNFerade	No	8.8	
	Yes	11.3	0.996
CA 19–9 pre-CRT	<90 U/mL	8.8	
	≥90 U/mL	8.8	0.626
CA 19–9 post-CRT minimum	<90 U/mL	12.3	
	≥90 U/mL	8.8	0.012

(3D = three dimensional conformal radiotherapy; CA 19–9 = carbohydrate antigen 19–9; CRT = chemoradiation; IMRT = intensity modulated radiotherapy; RT = radiotherapy).

**Figure 1:**
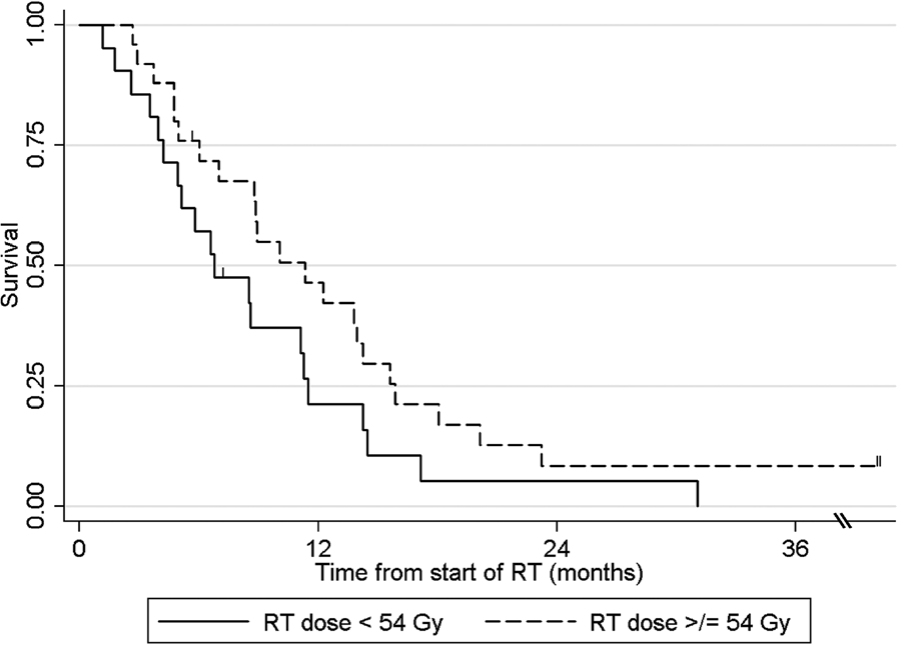
**Overall survival for patients treated with < or ≥ 54 Gy of radiotherapy.** Note: two long term survivors are censored at 58 and 88 months.

**Figure 2:**
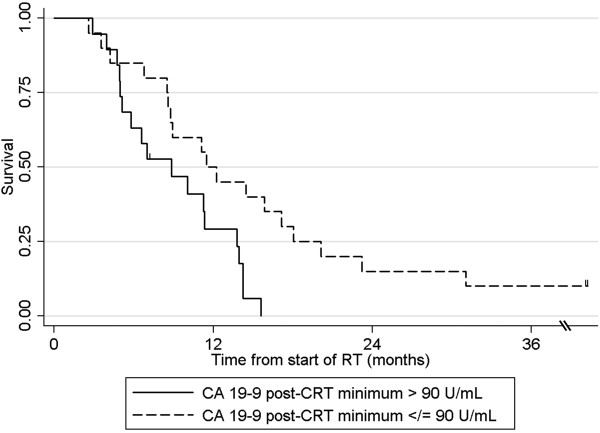
Overall survival for patients with CA 19–9 post-CRT minimum ≥ or < 90 U/mL.

**Figure 3:**
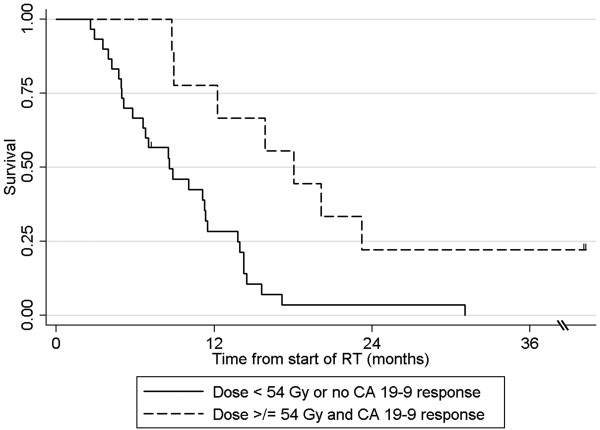
Overall survival for patients treated with ≥54 Gy of radiotherapy and a CA 19–9 post-CRT minimum < 90 U/mL versus all other.

On multivariable analysis, delivered RT dose ≥54 Gy (p = 0.028) and CA 19–9 post-CRT minimum <90 U/mL (p = 0.008) were associated with overall survival (Figures 1, 2; Table 4). Patients who were treated to a total dose ≥54 Gy and who experienced a post-CRT CA 19–9 minimum <90 U/mL had significantly longer survival than all other patients (median 18.0 versus 8.6 months, p = 0.002; Figure 3). A subset multivariable analysis excluding patients who did not complete RT (n = 5) showed that post-CRT CA 19–9 minimum <90 U/mL remained significantly associated with overall survival (p = 0.008) and delivered RT dose ≥54 Gy continued to trend towards improved overall survival (p = 0.065).

**Table 4:** Multivariable analysis of factors associated with overall survival

	**HR (95% CI)**	**p value**
Delivered RT dose ≥54 Gy	0.47 (0.24-0.92)	0.028
CA 19–9 nadir <90 U/mL	0.35 (0.17-0.76)	0.008

(CA 19–9 = carbohydrate antigen 19–9; CI = confidence interval; HR = hazard ratio; RT = radiotherapy).

Given that an improvement in overall survival with higher radiation dose would be attributable to improved local control, an exploratory analysis of the effect radiation dose on local failure was done. There were 5 local failures in the <54 Gy patients and 9 local failures in the ≥54 Gy patients (p = 0.82).

A recent study demonstrated improved outcomes in patients with CA 19–9 levels <100 U/mL pre-CRT [11]. We therefore performed an exploratory subset analysis of the effect of RT dose on survival in those patients who had a pre-CRT CA 19–9 <90 U/mL (2.5x the upper limit of normal). Median survival was higher for patients who received a RT dose ≥54 Gy (12.3 versus 3.6 months), but this difference was not statistically significant (p = 0.18), related to the limited number of patients in this subset.

## Discussion

The role for radiotherapy in the treatment of unresectable pancreatic cancer is controversial. For patients with non-metastatic unresectable pancreatic cancer, data support the use of concurrent 5-fluorouracil and radiotherapy with an improvement in overall survival [12]. Two randomized studies also show a survival benefit to CRT over chemotherapy alone [8,9]. However, other trials have failed to show a benefit to the addition of radiotherapy to chemotherapy [4]. In this single-institution review of patients with non-metastatic unresectable pancreatic adenocarcinoma treated with definitive CRT, the poor outcomes associated with this disease are observed. Our survival results are comparable to historical trials [12]. We demonstrate a dose–response relationship, with patients treated to ≥54 Gy surviving significantly longer than those patients treated to <54 Gy. Analysis of the two groups stratified by dose does not suggest that this improvement is merely due to an imbalance of prognostic factors.

The randomized trials for unresectable pancreatic cancer during the CT-imaging era used radiotherapy doses between 50.4 and 60 Gy. The previously tested dose of 60 Gy was only studied with split-course radiotherapy, which decreases the biological effectiveness in comparison to continuous treatment. The data presented suggest that with higher doses of continuous radiotherapy, the benefit of radiotherapy may be more substantial. Methods to increase the radiotherapy dose delivered to the pancreas have included intra-operative radiotherapy (IORT), stereotactic body radiotherapy (SBRT), intensity modulated radiotherapy (IMRT), and image-guided radiotherapy (IGRT). However, none of the methods have been evaluated in phase III trials.

Long-term survivors have been reported in a study using IORT [13]. IMRT has also been demonstrated to decrease the dose to critical structures without compromising tumor control [14]. A recent study investigating radiotherapy dose escalation using IMRT and active breathing control with gemcitabine showed promising 2-year freedom from local progression and overall survival of 59% and 30%, respectively [15]. SBRT is also a promising technique for dose escalation. In one study of IMRT with a SBRT boost to the primary tumor, local control was seen in 15/16 patients until death [16]. Results with primary SBRT have been mixed, with good rates of local control but significant toxicity and distant failure resulting in survival rates that are not superior to historical controls [17-21]. Further improvement of techniques such as these may allow for higher doses of radiotherapy to be delivered to the tumor while sparing normal tissue, thereby increasing the therapeutic ratio of radiotherapy. Our institution currently has an ongoing SBRT dose escalation protocol for unresectable pancreatic cancer.

In our current study, we demonstrated an improvement in overall survival with higher radiation dose, but were unable to show a corresponding improvement in local control with higher radiation dose. Given that our medical center is a tertiary referral center, many patients return to their local institutions after treatment for follow-up imaging. Therefore, the local and distant failure data are less robust that the survival data. In addition, it is important to emphasize that although the crude rate of local failure was higher in the ≥54 Gy patients, these patients were followed for slightly longer periods of time and may have had a greater opportunity to define local failure. This observation may explain why this difference was not significant on actuarial analysis.

Recent studies have examined induction chemotherapy followed by concurrent CRT as a method to select those patients most likely to benefit from aggressive local treatment with concurrent CRT [7,22,23]. These studies show improved outcomes in patients who complete induction chemotherapy and subsequently receive CRT. The GERCOR study treated patients to 55 Gy with 45 Gy administered in 1.8 Gy fractions daily and an additional 1.25 Gy four days per week for the final two weeks as a second daily dose [7]. Another study reported preliminary results from 28 patients treated with induction chemotherapy followed by 44.25 Gy in 15 fractions with concomitant capecitabine and demonstrated an 18 month overall survival of 63% [24]. Patients who do not progress through neoadjuvant chemotherapy are selected as a biologically favorable group without the propensity for early progression of metastases; the rate of distant metastasis after induction chemotherapy is approximately 30% in the two prospective trials. Radiotherapy dose escalation therefore may be able to further improve outcomes in patients not already harboring micrometastatic disease, as local therapy would presumably have a relatively higher impact in these patients. Review of the patterns of failure for the patients completing induction chemotherapy and CRT in the UCSF prospective study [22] show that 33% failed locally as first site of failure. Radiotherapy dose escalation for this subset of patients may delay or prevent recurrence, and thereby improve survival. Another possibility would be to test tumor tissue obtained at the time of biopsy for radiosensitivity, similar to a recent study that evaluated sensitivity to gemcitabine in vitro [25], to select patients most likely to benefit from aggressive local therapy.

Data from borderline resectable patients proceeding to curative resection also supports radiation dose escalation. The University of Virginia treated 8 patients with 50.4 Gy in 28 fractions and concurrent capecitabine and 8 patients with a hypofractionated regimen (50 Gy delivered in 20 fractions) and concurrent capecitabine prior to surgery. 50 Gy in 20 fractions offers a higher biologically equivalent radiation dose. Five of the 8 patients who underwent resection showed a >90% pathologic complete response in the hypofractionated group versus 1 of 8 patients in the standard fractionation group, suggesting that dose escalation with hypofractionation improves pathologic response [26].

We also demonstrated that a CA 19–9 minimum <90 U/mL (2.5x the upper limit of normal) is associated with improved survival. Prior studies have demonstrated that CA 19–9 levels can predict response to CRT and can be prognostic for outcome in the unresectable setting [11,25,27,28]. Yoo *et al.*[11] recently demonstrated that a CA 19–9 level <100 U/mL before or after CRT for unresectable pancreatic cancer is prognostic for overall survival. Our finding that a CA 19–9 level <90 U/mL after CRT supports the finding that a low CA 19–9 level after CRT for unresectable pancreatic cancer is prognostic for survival. It has been shown in the resectable setting that patients with high pre-operative CA 19–9 levels who experience normalization of their post-operative CA 19–9 levels had similar outcomes to those patients with low pre-operative CA 19–9 levels [29]. This suggests that CA 19–9 response in the definitive CRT setting may be a surrogate for local tumor control which in turn may predict for improved survival. We did not find an association between pre-treatment CA 19–9 and survival. However, we did demonstrate a trend towards improved survival with a delivered RT dose >54 Gy in patients who had a pre-CRT CA 19–9 <90 U/mL, suggesting that RT dose escalation in patients with lower tumor burden [11] may improve outcomes. Given the small number of patients in our subset, the difference in survival did not reach statistical significance.

This study has several limitations. First, this is a retrospective series subject to selection bias, in which the more favorable outcomes of patients treated to higher RT dose could potentially be due to uncontrolled factors and an improved ability to complete therapy. Although higher RT dose continued to trend towards improved survival upon exclusion of patients with incomplete courses of therapy, we are unable to exclude the possibility of a bias that more favorable patients were prescribed, or could tolerate, higher doses. Additionally, many patients diagnosed with locally advanced pancreatic cancer develop systemic metastases or progressive local disease and are not referred for radiotherapy. Therefore, the patients analyzed here may represent a selected group of patients with locally advanced pancreatic cancer. We were unable to include a report of toxicity data due to a lack of reliable grading of toxicity and limited follow-up. However, in those patients with >1 year of follow-up before death, there did not appear to be an increase in late toxicity in the high versus low radiotherapy dose groups. Also, the two long term survivors, both treated with doses of 59.4 Gy, are alive without grade 2+ gastrointestinal toxicity. Nevertheless, the risk for increased toxicity should be carefully weighed against the potential for improved survival when considering dose escalated RT.

In conclusion, patients treated with CRT as definitive treatment for unresectable pancreatic cancer generally had low survival. However, our data suggest that patients treated with ≥54 Gy or who experienced a post-CRT CA 19–9 minimum <90 U/mL had an improved likelihood of long-term survival. The results presented herein support the use of dose-escalated radiotherapy combined with systemic chemotherapy as a component of definitive treatment for unresectable pancreatic cancer. We are hopeful that through a combination of improving systemic chemotherapy, novel biologic agents, and targeted local therapy including dose-escalated radiotherapy, outcomes for patients with unresectable pancreatic cancer may improve.

## Abbreviations

CA 19–9: Carbohydrate antigen 19–9; CI: Confidence interval; CT: Computed tomography; CRT: Chemoradiation; GERCOR: Groupe cooperatéur multidisciplinaire en oncologie (French); Gy: Gray; IMRT: Intensity modulated radiation treatment; MRI: Magnetic Resonance Imaging; RT: Radiotherapy; SBRT: Stereotactic body radiotherapy. Presented at the 51st Annual Meeting of the American Society for Radiation Oncology, San Diego, California, November, 2010.

## Competing interests

Dr. Bruce Minsky is a consultant to Roche Pharmaceuticals and Varian Medical Systems. The other authors declare they have no competing interests.

## Authors’ contributions

DWG participated in the conception and design of the study, data acquisition, analysis and interpretation of data, and drafted the manuscript. CJN participated in data acquisition, analysis and interpretation of data, and helped to draft the manuscript. BDM participated in analysis and interpretation of data and helped to draft the manuscript. SLL participated in the conception and design of the study, data acquisition, analysis and interpretation of data, and helped to draft the manuscript. All authors read and approved the final manuscript.
